# Development of an antibiotic marker-free platform for heterologous protein production in *Streptomyces*

**DOI:** 10.1186/s12934-017-0781-y

**Published:** 2017-09-26

**Authors:** Laura Sevillano, Margarita Díaz, Ramón I. Santamaría

**Affiliations:** Instituto de Biología Funcional y Genómica (IBFG), Consejo Superior de Investigaciones Científicas/Universidad de Salamanca, C/Zacarías González no 2, 37007 Salamanca, Spain

**Keywords:** Heterologous protein expression, Antibiotic marker-free, *Streptomyces*, Toxin-antitoxin, Separate-component-stabilization system

## Abstract

**Background:**

The industrial use of enzymes produced by microorganisms is continuously growing due to the need for sustainable solutions. Nevertheless, many of the plasmids used for recombinant production of proteins in bacteria are based on the use of antibiotic resistance genes as selection markers. The safety concerns and legal requirements surrounding the increased use of antibiotic resistance genes have made the development of new antibiotic-free approaches essential.

**Results:**

In this work, a system completely free of antibiotic resistance genes and useful for the production of high yields of proteins in *Streptomyces* is described. This system is based on the separation of the two components of the *yefM/yoeBsl* (antitoxin/toxin) operon; the toxin (*yoeBsl*) gene, responsible for host death, is integrated into the genome and the antitoxin gene (*yefMsl*), which inactivates the toxin, is located in the expression plasmid. To develop this system, the toxin gene was integrated into the genome of a strain lacking the complete operon, and the antibiotic resistance gene integrated along with the toxin was eliminated by Cre recombinase to generate a final host strain free of any antibiotic resistance marker. In the same way, the antibiotic resistance gene from the final expression plasmid was removed by Dre recombinase. The usefulness of this system was analysed by checking the production of two hydrolases from different *Streptomyces*. Production of both proteins, with potential industrial use, was high and stable over time after strain storage and after serial subcultures. These results support the robustness and stability of the positive selection system developed.

**Conclusions:**

The total absence of antibiotic resistance genes makes this system a powerful tool for using *Streptomyces* as a host to produce proteins at the industrial level. This work is the first *Streptomyces* antibiotic marker-free system to be described.

**Graphical abstract:**
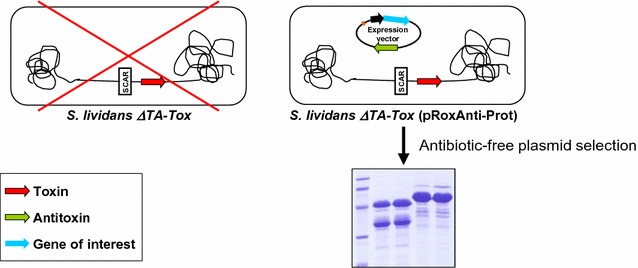
Antibiotic marker-free platform for protein expression in *Streptomyces*. The antitoxin gene present in the expression plasmid counteracts the effect of the toxin gene in the genome. In absence of the expression plasmid, the toxin causes cell death ensuring that only plasmid-containing cells persist.

## Background

The use of living organisms to produce commercially important value-added biomolecules is of great importance for the different industrial sectors of society such as agriculture, food, energy and pharmaceutical. Recently, Zhang et al. classified the use of organisms, called biomanufacturing, into four historical revolutions. Heterologous protein production, also called biologics [1], corresponds to what is known as biomanufacturing 3.0. This type of technology began in the 1980s with the production of large-size proteins like erythropoietin, insulin, among others, from genetically modified microorganisms [2] thanks to the development of recombinant DNA and advanced cell culture techniques.

Many of the proteins produced by this DNA recombinant technology are hydrolytic enzymes like amylases, cellulases, hemicellulases, lipases and proteases, which are important products for many different types of industries such as food and beverages, textile, detergent, pharmaceuticals [3], animal feed [4], biofuels and fine-chemical industries, among others. Presently, approximately 90% of industrial enzymes are recombinant versions produced in bacteria and fungi [5], and their use is expected to increase due to a growing need for sustainable solutions. Moreover, the discovery of new enzymes to add to the currently available toolbox and the development of optimized strategies for the production of these hydrolytic enzymes is a central goal of many industrial sectors [6].

There is a wide spectrum of hosts used as expression systems for the production of recombinant proteins that include bacteria, yeast, filamentous fungi, insect and mammalian cells and whole transgenic plants and animals [7]. Although the use of each host has different advantages and disadvantages, their main objective is process optimisation [7]. The most commonly used host to produce recombinant proteins is *E. coli* owing to its easy and rapid growth. However, the use of *E. coli* does present several drawbacks which can be overcome by the use of other types of host. Gram-positive hosts, such as *Streptomyces*, are an excellent alternative due to high secretion efficiency, which makes their use feasible for the direct release of proteins of interest into the culture medium. This in turn facilitates downstream procedures, such as extraction and purification, and consequently decreases the costs associated with the production of recombinant proteins. In addition, some *Streptomyces* strains have a relatively low level of endogenous extracellular proteolytic activity in comparison with other hosts [8, 9]. Also, *Streptomyces* has proved useful to produce high levels of different proteins [8–12].

The use of enzymes produced by microorganisms as biocatalysts in industry can be considered “green chemistry” because no toxic waste is generated [6]. Nevertheless, many of the plasmids used for recombinant production of enzymes are based on the use of antibiotic resistance genes as selection markers. Due to the intensifying problem of the appearance of antibiotic resistant strains [13–15] and more legal requirements, it is important to avoid the use of these antibiotics as much as possible in the industrial process. As regards different antibiotic-free approaches have been developed, which include the complementation of auxotrophic bacterial strains, toxin-antitoxin-based systems, operator-repressor titration, RNA-based selection markers and the overexpression of essential genes. These alternative selection systems are summarized in Vandermeulen et al. [16].

Toxin-antitoxin systems (TAs) are ubiquitous in plasmids and genomes of bacteria and archaea, and are small genetic modules composed of a biologically active protein molecule (toxin) and the corresponding inhibitor (antitoxin). Many functions have been assigned to TAs like DNA stabilization, stress response, persistence and protection against mobile genetic elements [17–20]. The efficiency of these TAs depends on the different stability of both components, where toxins are highly resistant to proteases and antitoxins are more labile and readily degraded by proteases.

In view of this, our group developed a separate component stabilization (SCS) system for *Streptomyces lividans* based on the *yefM/yoeBsl* toxin-antitoxin operon [21]. In this system the toxin (*yoeBsl*) gene, responsible for host death, is integrated into the genome, and the antitoxin (*yefMsl*) gene which inactivates the toxin is located in the expression plasmid [22]. To develop this system, the toxin (*yoeBsl*) gene was integrated into the genome of a strain lacking the complete operon, and with the antitoxin (*yefMsl*) gene carried by a thermosensitive plasmid [strain: *S. lividans ∆TA* (pGM160-YefMsl^ts^)] [22]. This strain was the starting point for the improvement of the *Streptomyces* SCS expression system, as described in the Results section. After toxin integration, the expression plasmids with the gene(s) of interest and the antitoxin are introduced into the host strain [*S. lividans ∆TA*-*pKC796*-*Tox* (pGM160-YefMsl^ts^)] in place of the thermosensitive plasmid, and ultimately used for heterologous protein production. Thus, the antitoxin counteracts the effect of the toxin when the expression plasmid is present in the cell. By contrast, when the plasmid is lost the toxin causes cell death, ensuring that only the plasmid-containing cells persist [22]. Using this system, stable and high proteins yields, without the addition of antibiotics into the production process, was achieved [22]. This SCS strategy has also been shown to be effective in *E. coli* [23–26].

Additionally, it must be noted that although this SCS system does not require the addition of antibiotics to maintain the stable production of enzymes of interest, the antibiotic markers are still present in the host strain and in the expression plasmids used. These antibiotic resistance genes comprise a potential risk for antibiotic resistance transmission by horizontal gene transfer [27–29].

**Fig. 1:**
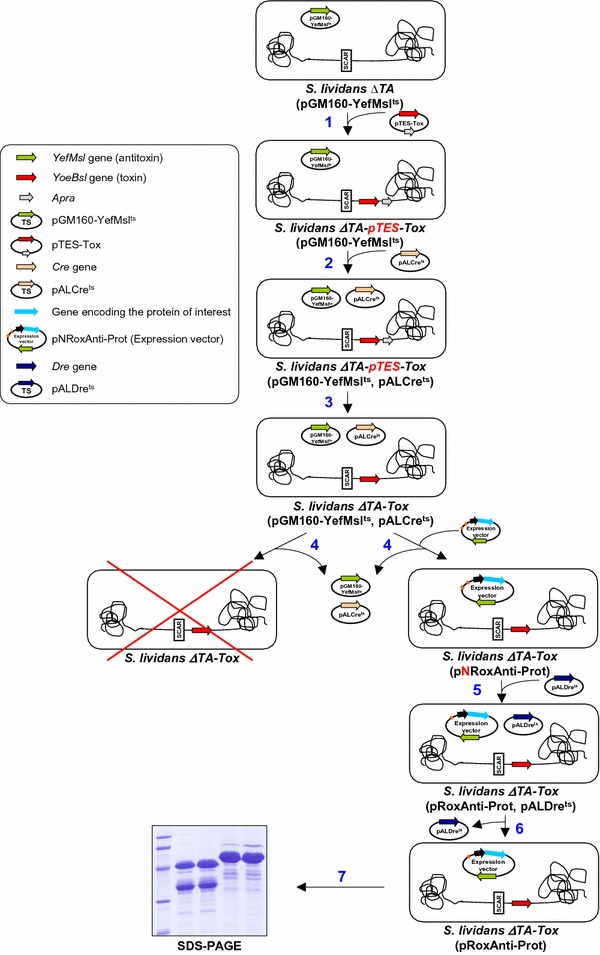
Diagram of the separate component-stabilization system in *Streptomyces*. 1. Integration of the toxin (*yoeBsl*) gene into the chromosome of the *S. lividans ∆TA* (pGM160-YefMsl^ts^) strain [22] with plasmid pTES-Tox. 2. Transformation with pALCre^ts^ to generate *S. lividans ∆TA*-*pTES*-*Tox* (pGM160-YefMsl^ts^, pALCre^ts^). 3. Elimination of apramycin resistance gene by induction of Cre recombinase. 4. Transformation with the expression plasmid (pNRoxAnti-Prot) and removal of the temperature-sensitive plasmids. 5. Transformation with pALDre^ts^ and elimination of the neomycin resistance gene from the expression plasmid (pNRoxAnti-Prot) by Dre recombinase induction. 6. Removal of the temperature-sensitive plasmid pALDre^ts^ and generation of the final host strain [*S. lividans ∆TA* -*Tox* (pRoxAnti-Prot)]. 7. Protein production

In this work, we developed an optimized version of the *S. lividans* SCS expression platform previously reported [22]. Both, the apramycin resistance gene from the host strain and the neomycin resistance gene from the expression plasmids have been removed to obtain a completely antibiotic marker-free system. To this end, two site-specific recombinases (SSRs) Cre and Dre were used to construct the host strain and the expression plasmids. Although SSR methodology has been applied in actinomycete genome manipulation for more than 20 years [30], it is only recently that the use of new SSRs has been optimized in these microorganisms, broadening their possible application [30–33]. The strategy followed within this work is summarized in Fig. 1.

With this antibiotic marker-free system, the production of two proteins, the α-amylase Amy from *S. griseus* IMRU3570 [34] and the xylanase Xys1 from *S. halstedii* JM8 [35], was high and stable over time, validating the efficiency of this new platform for heterologous production of proteins in *S. lividans*. To our knowledge this is the first complete antibiotic marker-free system developed for *Streptomyces*. Thus, the total absence of antibiotic resistance genes presents an interesting potential use of this system in industry.

## Results and discussion

### Construction of an antibiotic marker-free host strain

**Fig. 2:**
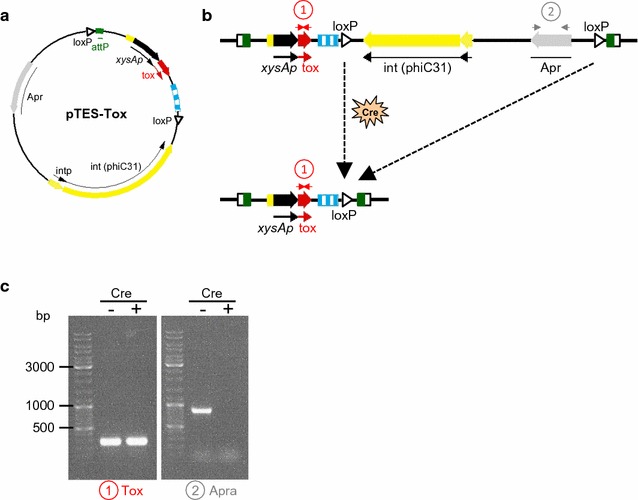
Elimination of the apramycin resistance gene in the host strain genome. **a** Diagram of the integrative pTES-Tox plasmid. **b** Diagram of the pTES-Tox plasmid backbone deletion by the action of Cre recombinase (modified from [33]). **c** PCR amplification of the toxin gene (Tox) with primers LS-008 and LS-009 (1) and the apramycin resistance gene (Apra) with primers LS-113 and LS-114 (2) from *S. lividans* genomic DNA before, *S. lividans ∆TA*-*pTES*-*Tox* (−), and after, *S. lividans ∆TA*-*Tox* (+), Cre activity

As mentioned above, in a previous work, the strain *S. lividans ∆TA*-*pKC796*-*Tox* (pGM160-YefMsl^ts^) was used as a host to produce high levels of proteins without the use of antibiotics during the production step [22]. However, to obtain a completely antibiotic marker-free system it is necessary to delete the apramycin gene integrated into the genome during the generation of the strain. With this purpose in mind, a new plasmid pTES-Tox (Fig. 2a and Table 2) was used to integrate the toxin gene (*yoeBsl*) into the genome of the strain *S. lividans ∆TA* (pGM160-YefMsl^ts^) [22]. In this new plasmid the toxin gene and the phage attachment site (*attP*) are flanked by the target sites for the Cre recombinase (*loxP*) (Fig. 2a).

*Streptomyces lividans ΔTA* (pGM160-YefMsl^ts^) strain [22], which harbours the temperature sensitive plasmid pGM160-YefMsl^ts^ with the antitoxin gene (*yefMsl*) [22], was transformed with pTES-Tox generating the strain *S. lividans ∆TA*-*pTES*-*Tox* (pGM160-YefMsl^ts^) (step 1 in Fig. 1). Then, this strain was transformed with the plasmid pALCre^ts^ [33] encoding the Cre recombinase yielding *S. lividans ∆TA*-*pTES*-*Tox* (pGM160-YefMsl^ts^, pALCre^ts^) strain (step 2 in Fig. 1). Afterwards, the induction of this Cre recombinase allowed the plasmid backbone between the two *loxP* sites to be deleted, leaving only the toxin gene integrated in the genome (Fig. 2b). Successful elimination of the apramycin resistance gene was checked by PCR (Fig. 2c). The strain obtained after the elimination of apramycin gene was designated *S. lividans ∆TA*-*Tox* (pGM160-YefMsl^ts^, pALCre^ts^) (step 3 in Fig. 1) and was used as the final antibiotic marker-free host to produce heterologous proteins in *S. lividans*.

### Effectiveness of the antibiotic marker-free host strain to produce high levels of protein

The usefulness of this new strain *S. lividans ∆TA*-*Tox* (pGM160-YefMsl^ts^, pALCre^ts^), without apramycin resistance in its genome, was analysed by checking the production of two proteins: the Amy α-amylase from *S. griseus* IMRU3570 [34] and the Xys1 xylanase from *S. halstedii* JM8 [35]. *S. lividans wt* and *S. lividans ∆TA* strains were used as controls.

**Fig. 3:**
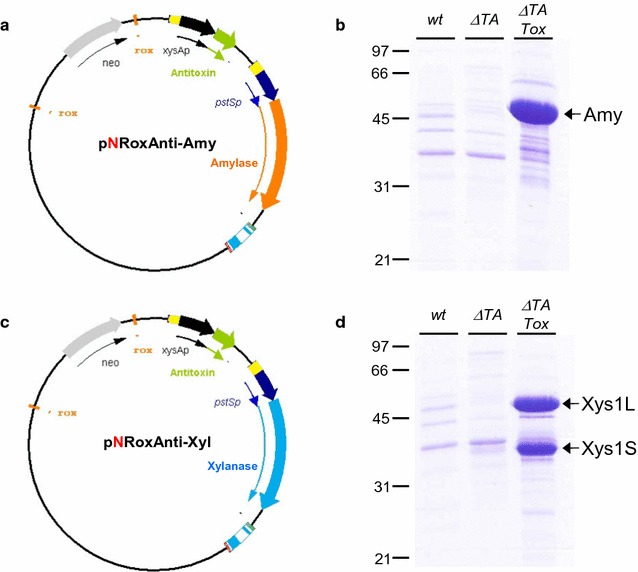
Amylase and Xylanase production by the different strains of *S. lividans.* **a**, **c** Diagram of the expression plasmids pNRoxAnti-Amy and pNRoxAnti-Xyl. **b**, **d** Amylase (**b**) and xylanase (**d**) production by *S. lividans wt*, *S. lividans ΔTA* and *S. lividans ΔTA*-*Tox* transformed with pNRoxAnti-Amy (**b**) and pNRoxAnti-Xyl (**d**) after 6 days of culture in YES medium supplemented with 3% xylose. 10 µL of the supernatant was loaded into each track

Two new expression plasmids pNRoxAnti-Amy and pNRoxAnti-Xyl were generated (Fig. 3a, c). These plasmids harbour the antitoxin (*yefMsl*) gene under the control of the xylanase promoter *xysAp* [35] and the Amy α-amylase or the Xys1 xylanase, respectively, under the control of *pstSp* promoter from *S. lividans* [36]. These plasmids were generated with the target sites for the Dre recombinase (*rox*) flanking the neomycin resistance gene for its subsequent elimination (see below). In general, the expression plasmid generated was named pNRoxAnti-Prot (Fig. 1).

*Streptomyces lividans ∆TA*-*Tox* (pGM160-YefMsl^ts^, pALCre^ts^) and the control strains (*S. lividans wt* and *S. lividans ∆TA*) were transformed with pNRoxAnti-Amy or pNRoxAnti-Xyl (step 4 in Fig. 1). After removal of the temperature sensitive plasmids (pGM160-YefMsl^ts^ and pALCre^ts^) by incubation at 37 °C, the transformants were cultured at 28 °C in liquid YES medium without antibiotic and supplemented with 3% xylose to induce both promoters. The production of amylase or xylanase was analysed by SDS-PAGE of the supernatants collected after 6 days of culture (Fig. 3b, d).

A high production of amylase or xylanase was observed with the *S. lividans ∆TA*-*Tox* strain; however, no amylase and xylanase production was observed in the control strains (*S. lividans wt* and *S. lividans ∆TA*) (Fig. 3b, d). These results indicate that the toxin gene in the genome of the *S. lividans ∆TA*-*Tox* strain was exerting a positive selection for the maintenance of the expression plasmids containing the antitoxin, since the loss of the latter results in cell death. In contrast, in absence of antibiotic selection pressure, the expression plasmids (pNRoxAnti-Amy or pNRoxAnti-Xyl) present in the control strains (*S. lividans wt* and *S. lividans ∆TA*) were lost and consequently no protein production was observed (Fig. 3b, d).

Therefore, the new strain (*S. lividans ∆TA*-*Tox*) without apramycin resistance allowed positive plasmid selection in the same way as the previously reported (*S. lividans ∆TA*-*pKC796*-*Tox*) [22] but without the potential problems associated with the remaining presence of an antibiotic resistance gene in the host strain.

### Elimination of the antibiotic marker gene in the expression plasmid and analysis of protein production

Nevertheless, it is still necessary to eliminate the neomycin resistance gene present in the expression plasmids used (pNRoxAnti-Amy or pNRoxAnti-Xyl) in order to generate a completely antibiotic marker-free system. This antibiotic resistance gene was necessary to make the constructions in *E. coli* but was subsequently eliminated in *Streptomyces*. To facilitate the deletion of the antibiotic marker gene, the target sites for Dre recombinase (*rox*) [33] were introduced flanking the neomycin resistance gene as mentioned before.

**Fig. 4:**
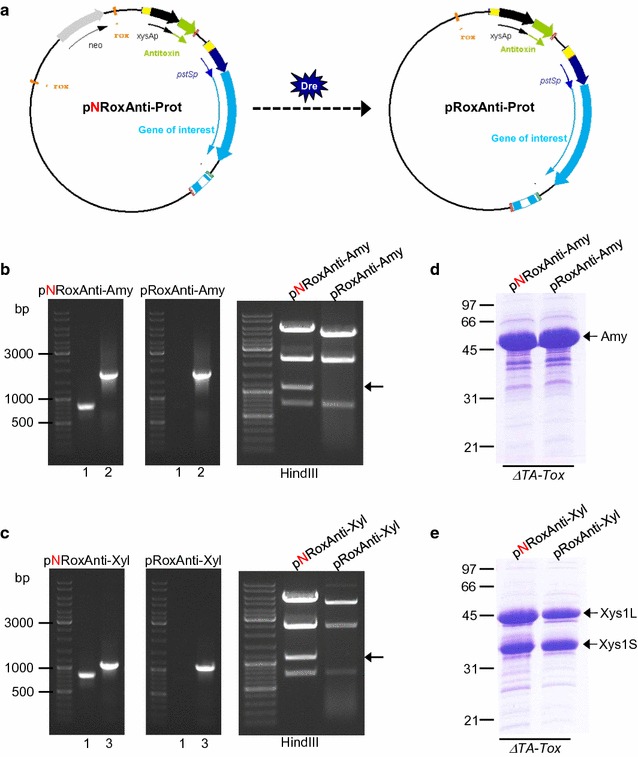
Elimination of Neomycin gene in the expression plasmid. **a** Diagram of the deletion of neomycin resistance gene from the expression plasmid by Dre recombinase. **b**, **c** PCR and restriction analysis of pNRoxAnti-Amy and pRoxAnti-Amy (**b**) or pNRoxAnti-Xyl and pRoxAnti-Xyl (**c**). 1. Neo: Primers LS124 and LS125. 2. Amy: Primers MRG11 and MRG12. 3. Xyl: Primers LS116 and LS117. The arrow shows the neomycin restriction band. **d**, **e** Amylase and xylanase production by *S. lividans ΔTA*-*Tox* transformed with pNRoxAnti-Amy and pRoxAnti-Amy (**d**) or pNRoxAnti-Xyl and pRoxAnti-Xyl (**e**) after 6 days of culture in YES medium supplemented with 3% xylose. 10 µL of the supernatant was loaded into each track

The transformation of the strains *S. lividans ∆TA*-*Tox* (pNRoxAnti-Amy) and *S. lividans ∆TA*-*Tox* (pNRoxAnti-Xyl) with the plasmid pALDre^ts^ [33] (step 5 in Fig. 1) resulted in the deletion, by Dre recombinase, of the neomycin gene flanked by the *rox* sites generating the plasmids pRoxAnti-Amy or pRoxAnti-Xyl. This plasmid lacking the neomycin resistance gene was called pRoxAnti-Prot (Fig. 4a).

In order to check the effective deletion of the neomycin resistance gene, after the elimination of the pALDre^ts^ plasmid (step 6 in Fig. 1), the plasmids pRoxAnti-Amy and pRoxAnti-Xyl were recovered from the corresponding *S. lividans ∆TA*-*Tox* strains and the elimination of the neomycin gene was confirmed by PCR and by restriction analysis (Fig. 4b, c).

The amount of protein produced after the elimination of the marker gene was analysed and compared to that produced with the parental plasmids harbouring the neomycin resistance gene. Transformants of *S. lividans ∆TA*-*Tox* (pRoxAnti-Amy) or *∆TA*-*Tox* (pRoxAnti-Xyl) and transformants of *S. lividans ∆TA*-*Tox* (pNRoxAnti-Amy) or *∆TA*-*Tox* (pNRoxAnti-Xyl) were cultured for 6 days at 28 °C in liquid YES medium without antibiotic, and supplemented with 3% xylose. The production of amylase or xylanase was analysed by SDS-PAGE using the collected supernatants (Fig. 4d, e). The amount of amylase and xylanase produced after the elimination of the neomycin resistance gene was similar to that obtained with the neomycin resistant plasmids (Fig. 4d, e).

At the end of this process, a new expression platform for *Streptomyces* completely free of antibiotic resistance genes was developed and suitable to produce proteins at industrial level.

### Plasmid stability

A robust system for producing proteins at the industrial level must have a high protein yield and also be able to maintain stable production. Stability of the developed antibiotic marker-free platform was analysed under different conditions such as long-term culturing, after the strains were stored as frozen mycelia or as frozen spores and after serial subcultures.

**Fig. 5:**
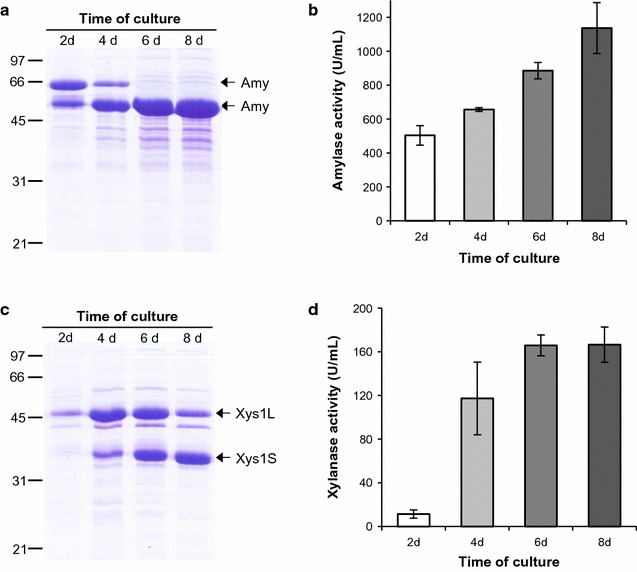
Enzyme production over the time of culture. **a**, **c** Amylase and xylanase production by *S. lividans ΔTA*-*Tox* transformed with pRoxAnti-Amy (**a**) and pRoxAnti-Xyl (**c**) after 2, 4, 6 and 8 days of culture in YES medium supplemented with 3% xylose. 10 µL of the supernatant was loaded into each track. **b**, **d** Amylase (**b**) and xylanase (**d**) activity of the supernatants. The histogram bars are the means of three experiments

Initially, plasmid stability in long-term cultures was analyzed by measuring the enzymatic activity in the supernatants of *S. lividans ∆TA*-*Tox* (pRoxAnti-Amy) and *S. lividans ∆TA*-*Tox* (pRoxAnti-Xyl) at different culture times (Fig. 5). An increase in enzyme production and activity were observed during the time of culture. After 8 days, high enzyme production was observed, suggesting that the cells still contained the plasmids. The processing of both proteins over time was also observed (Fig. 5), as has been previously described [34, 37].

**Fig. 6:**
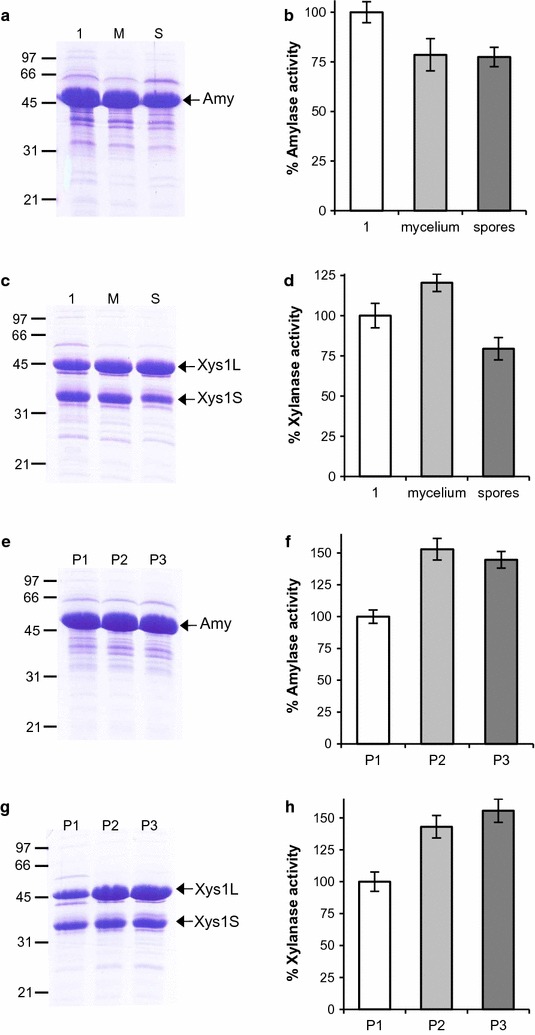
Enzyme production after strains storage and after serial subcultures. **a** and **c** Amylase and xylanase production by *S. lividans ΔTA*-*Tox* transformed with pRoxAnti-Amy (**a**) and pRoxAnti-Xyl (**c**) after mycelia storage (M) and after sporulation (S). 10 µL of the supernatant collected after 6 days was loaded into each track. **b** and **d** Percentages of amylase (**b**) and xylanase (**d**) activity of the supernatants compared with the original culture (1). **e** and **g** Amylase and xylanase production by *S. lividans ΔTA*-*Tox* transformed with pRoxAnti-Amy (**e**) and pRoxAnti-Xyl (**g**) after three 100-fold serial dilutions every 2 days (P1, P2, and P3) in fresh YES medium supplemented with 3% xylose. 10 µL of supernatants collected after 6 days was loaded into each track. **f** and **h** percentage of amylase (**f**) and xylanase (**h**) activity of the supernatants. The histogram bars are the mean of three experiments

*Streptomyces* strains can be stored for long periods of time by freezing mycelia or spores in 20% glycerol. To check plasmid stability after mycelia freezing, 100 µL of frozen mycelia suspension, were inoculated into 10 mL of YES medium with 3% of xylose for 2 days. Then, 100 µL of the preculture were reinoculated in 10 mL of YES medium with 3% of xylose for 6 days at 28 °C. Amylase and xylanase activities in the supernatants were compared with the activities observed in the original cultures (Fig. 6b, d). Plasmid stability after sporulation was analyzed in a similar way, 5 × 10^5^ spores were inoculated in 10 mL of YES medium with 3% xylose for 6 days and enzyme production was analyzed by SDS-PAGE and by measuring enzymatic activity (Fig. 6b, d). More than 75% of enzymatic activity was obtained after the storage of the strains with two different methods. In the case of xylanase, the activity observed after freezing the mycelia was even greater than the original culture (Fig. 6d). These results again confirm the strong efficiency of our system to stably maintain the expression plasmids.

Finally, plasmid stability was assessed after three serial 100-fold dilutions (P1, P2 and P3) of cultures in fresh YES medium every 2 days. Using the same procedure as mentioned above, the amount of enzyme in the supernatant of each subculture was analyzed by SDS-PAGE after 6 days (Fig. 6e, g). Also enzymatic activity was measured (Fig. 6f, h) and compared with the activity obtained in the first culture of the series. It should be noted that there was even an enhanced production of both amylase and xylanase throughout the different passages, and as consequence an increase in the enzymatic activity of the supernatants.

All of these results support the robustness and stability of this improved selection system.

## Conclusions

In this work, a system that is completely free of antibiotic resistance genes and useful for the production of high yields of proteins in *Streptomyces* without the use of antibiotics as selective agents is reported.

This system is an improved version of the system previously described by our group, and is based on the separation of the two components of the *yefM/yoeBsl* (toxin/antitoxin) operon. The absence of antibiotic resistance genes brings additional value to this expression system and makes using *Streptomyces* as a host a powerful tool for the production of proteins that can be used within different industrial sectors.

## Methods

### Bacterial strains and growth conditions

The *E. coli* DH5α strain [38] was used for the cloning and isolation of plasmids. It was grown in Luria–Bertani (LB) liquid broth or on LB agar. All manipulations in *E. coli* were done following standard procedures [38, 39].

*Streptomyces lividans* 1326 and derivatives were grown on solid R2YE medium for transformation, on MSA medium for sporulation [40], and in liquid YES medium (1% yeast extract, 10.3% sucrose, 5 mM MgCl_2_) supplemented with 0.5% glucose and 0.5% glycine for collecting cells to make protoplasts, and YES medium supplemented with 3% xylose for protein expression. Liquid cultures were carried out in baffled flasks at 28 °C and 200 rpm. All manipulations in *Streptomyces* were done as indicated by Kieser [40].

### Apramycin gene deletion in the host strain

Protoplasts of *S. lividans ΔTA*-*pTES*-*Tox* (pGM160-YefMsl^ts^) [22] were transformed with pALCre^ts^ [33] and selected with 100 μg/mL hygromycin. Single colonies were reinoculated in patches on R2YE plates with 100 μg/mL hygromycin for 3 days and then cultured in TSB medium with 15 μg/mL thiostrepton for 3 days. Following on, serial dilutions of these cultures were streaked out to obtain single colonies on YEPD plates with 15 μg/mL thiostrepton. The single colonies were picked and streaked in parallel on YEPD agar and YEPD agar with 50 μg/mL apramycin to check for the loss of apramycin resistance. Elimination of apramycin resistance gene was checked by PCR from *S. lividans ∆TA*-*Tox* genomic DNA using primers LS-113 and LS-114 (Table 1), and the presence of the toxin (*yoeBsl*) gene in the genome was confirmed by PCR with primers LS008 and LS009 (Table 1).

**Table 1:** Oligonucleotides used

Name	Sequence 5′–3′	Use
LS-005	TTTTTTCATATGTCCATCACCGCCAGCGAAG	Forward (Fd) for *yefMsl* amplification. NdeI sequence is underlined
LS-007	TTTTTTAAGCTTCACGCCCGCTCCGCGTCCG	Reverse (Rev) for *yefMsl* amplification. HindIII sequence is underlined
LS-008	TTTTTTCATATGAGGATCACTTTCACGTCCCAC	Fd for *yoeBsl* amplification. NdeI sequence is underlined
LS-009	TTTTTTCTCGAGTCAGTAGTGGTAGCGCGCCTGG	Rev for *yoeBsl* amplification. XhoI sequence is underlined
LS-113	CGACTGATGTCATCAGCGGTGG	Fd for *aac(3′)IV* (apramycin resistance gene) amplification
LS-114	CCAACGTCATCTCGTTCTCCGC	Rev for *aac(3′)IV* (apramycin resistance gene) amplification
LS-124	ATGATTGAACAAGATGGATTGCACG	Fd for *aph(3′)* (neomycin resistance gene) amplification
LS-125	TCAGAAGAACTCGTCAAGAAGGCG	Rev for *aph(3′)* (neomycin resistance gene) amplification
MRG-11	TTTTTTCATATGGCCCGCAGACTCCGCACC	Fd for *amy* amplification. NdeI sequence is underlined
MRG-12	TTTTTTCTCGAGGCCGCGCCAGGTGTCGTTGAG	Rev for *amy* amplification. XhoI sequence is underlined
LS-116	CATATGGCTCAGAATCCCCCGG	Fd for *xysA* amplification
LS-117	CTCGAGCGCGGCGAGCACCG	Rev for *xysA* amplification

### Neomycin gene deletion in the expression plasmids

Transformation of the host strain with the expression plasmids and colony selection was done as described in Sevillano et al. [22]. Protoplasts of *S. lividans ∆TA*-*Tox* (pNRoxAnti-Amy) or *∆TA*-*Tox* (pNRoxAnti-Xyl) were transformed with pALDre^ts^ [33] and selected with 100 μg/mL hygromycin. Single colonies were reinoculated in patches on R2YE plates for 3 days and then streaked out on R2YE plates to obtain single colonies. The single colonies were picked and streaked in parallel on R2YE agar, R2YE agar with 15 μg/mL neomycin and R2YE agar with 100 μg/mL hygromycin to check for the loss of neomycin resistance and the loss of the pALDre^ts^ plasmid. Elimination of the neomycin resistance gene in the plasmid was checked by PCR using primers LS-124 and LS-125 (Table 1) and by restriction analysis.

### Plasmid constructions

#### pTES-Tox

Plasmid pN702Gem3-Tox [21] was digested with *Bgl*II, and the DNA fragment containing the *yoeBsl* (toxin) gene was ligated with pTES [33] (Table 2) digested with the same enzyme. In this plasmid, the toxin gene is regulated by the *xysA* promoter and is flanked by two transcriptional terminators. Two *loxP* sites are flanking the toxin gene and the phage attachment *attP* site (Fig. 2).

**Table 2:** Plasmids used

Plasmid	Characteristics	Reference
pGM160	*E. coli*/*Streptomyces* shuttle vector. Thiostrepton and gentamicin resistance	[44]
pGM160-YefMsl	pGM160 derivative. The *xysA* promoter from *S. halstedii* controls *yefMsl* expression	[21]
pN702Gem3	*E. coli*/*Streptomyces* shuttle vector. Neomycin resistance. High-copy number	[37]
pN702Gem3-Tox	pN702GEM3 derivative. The *xysA* promoter from *S. halstedii* controls toxin expression	[21]
pTES	*E. coli*/*Streptomyces* shuttle vector. Apramycin resistance. Integrative plasmid. *attP* flanked by *loxP* sites	[33]
pTES-Tox	pTES derivative. The *xysA* promoter from *S. halstedii* controls toxin expression	This work
pXHis1	pBluescript SK derivative. Ampicillin resistance. The *xysA* promoter from *S. halstedii* controls xylanase expression	[41]
pXHis1-Anti-T	pXHis1 derivative. The *xysA* promoter from *S. halstedii* controls antitoxin expression	This work
pTOS	*E. coli*/*Streptomyces* shuttle vector. Apramycin resistance. Integrative plasmid. *attP* flanked by *rox* sites	[33]
pTOS-Neo	pTOS derivative. The neomycin resistance gene is flanked by rox sites	This work
pN702Gem3Rox	pN702Gem3 derivative. The neomycin resistance gene is flanked by rox sites	This work
pN702Gem3Rox-Anti-T	pN702Gem3Rox derivative. The *xysA* promoter from *S. halstedii* controls antitoxin expression	This work
pNUF5	pN702Gem3 derivative. The *pstS* promoter from *S. lividans* controls xylanase expression	[36]
pNUF-Amy	pNUF5 derivative. The *pstS* promoter from *S. lividans* controls amylase expression	[22]
pNRoxAnti-Xyl	pN702Gem3Rox-Anti-T derivative. The *xysA* promoter from *S. halstedii* controls antitoxin expression and the *pstS* promoter from *S. lividans* controls xylanase expression	This work
pNRoxAnti-Amy	pN702Gem3Rox-Anti-T derivative. The *xysA* promoter from *S. halstedii* controls antitoxin expression and the *pstS* promoter from *S. lividans* controls amylase expression	This work
pRoxAnti-Xyl	pNRoxAnti-Xyl derivative without neomycin resistance gene	This work
pRoxAnti-Amy	pNRoxAnti-Amy derivative without neomycin resistance gene	This work

#### pN702Gem3Rox

Plasmid pN702Gem3 [37] was digested with *Blp*I, blunt ended with DNA polymerase klenow fragment and then digested with *Nhe*I. The DNA fragment containing the *aph(3′)* (neomycin resistance) gene was ligated into pTOS [33] digested with *Eco*RV and *Xba*I to obtain pTOS-Neo, which was used as an intermediate plasmid. To obtain pN702Gem3Rox, pTOS-Neo was digested with *Bsp*HI and *Hin*cII, and the DNA fragment containing the *aph(3′)* gene flanked by *rox* sites was blunt ended and ligated into pN702Gem3 digested with *Blp*I and *Nhe*I and blunt. In this plasmid two *rox* sites flank the neomycin resistance gene.

#### pN702Gem3Rox-Anti-T

*yefMsl* (antitoxin) was amplified by PCR from *S. lividans* 1326 genomic DNA using primers LS-005 and LS-007 (Table 1). The resulting fragment was digested with *Nde*I and *Hin*dIII and ligated into plasmid pXHis1, [41] (Table 2) digested with the same enzymes, to obtain plasmid pXHis1-Anti-T, which was used as an intermediate plasmid. Plasmid pN702Gem3Rox-Anti-T was obtained by digesting pXHis1-Anti-T with *Bgl*II, purifying the corresponding *yefMsl* band and ligating it into pN702Gem3Rox digested with the same enzyme. In this plasmid, the antitoxin gene is regulated by the *xysA* promoter and lacks the *fdt* transcriptional terminator at 3′ end.

#### pNRoxAnti-Xyl

This plasmid contains the ORF of the xylanase *xysA* gene from *S. halstedii* [35] under the control of the *S. lividans pstS* promoter, and the ORF coding for the antitoxin (*YefMsl*) under the control of the *xysA* promoter. The plasmid originated from the pNUF5 plasmid [36] which contains the xylanase gene regulated by *pstSp*; pNUF5 was digested with *Bsp*HI and *Nhe*I, and the *xysA* gene band was ligated into pN702Gem3Rox-Anti-T digested with *Bsp*HI and *Xba*I.

#### pNRoxAnti-Amy

This plasmid contains the ORF of the amylase gene from *S. griseus* [34] under the control of the *S. lividans pstS* promoter and the ORF coding for the antitoxin (*YefMsl*) under the control of the *xysA* promoter. To construct the plasmid, plasmid pNUF-Amy [22], which contains the amylase gene regulated by *pstSp*, was digested with *Bsp*HI and *Nhe*I and the corresponding band was ligated into pN702Gem3Rox-Anti-T digested with *Bsp*HI and *Xba*I.

### Sequence analyses

All constructions were sequenced on both strands using a Perkin Elmer ABI Prism 377 DNA sequencer. *In silico* plasmids were obtained with the Gene Construction Kit software (GCK, Textco).

### Protein analysis

Protein profiles were analysed by denaturing polyacrylamide gel electrophoresis (SDS-PAGE) in a MiniProtean II system (Bio-Rad). Proteins were detected by 0.5% Coomassie brilliant blue R staining and low molecular weight standards from Bio-Rad were used as markers.

### Xylanase and amylase activities assays

Xylanase activity was measured with the dinitrosalicylic acid (DNS) method [42] using xylose as standard. One unit of xylanase activity was defined as the amount of enzyme required to release 1 µmol of reducing sugars in 1 min (expressed as xylose equivalents). All data shown are means of at least three different experiments.

Amylase activity was measured with the method described by Xiao [43]. One unit of amylase activity was defined as the amount of enzyme required to degrade 1 mg of soluble starch in 1 h. All data shown are means of at least three different experiments.
